# Vaccine Narratives and Public Health: Investigating Criticisms of H1N1 Pandemic Vaccination

**DOI:** 10.1371/currents.outbreaks.17b6007099e92486483872ff39ede178

**Published:** 2015-02-25

**Authors:** Sudeepa Abeysinghe

**Affiliations:** University of Edinburgh

**Keywords:** vaccine hesitancy

## Abstract

Vaccine hesitancy is often understood and explored on the level of individual decision-making. However, questions surrounding the risk and efficacy of vaccination are evident in wider public discourse; social narratives of vaccination inform and impact on the individual level. This paper takes a narrative analysis approach from the sociology of health to examine data drawn from a wider study on global public health responses to the H1N1 pandemic. The paper concentrates upon criticisms to mass vaccination as recounted within the Council of Europe’s debate of the handling of H1N1. It shows that three narratives were particularly dominant: problematizing the use of vaccination as a public health response; criticising the efficacy of the vaccines; and, questioning the safety of the strategy. This debate presents an important case study in understanding the way in which vaccines are problematized within the public discourse.

## Related Articles

The article is part of the *PLOS Currents Outbreaks *"Vaccine Hesitancy Collection".

## Introduction

Mass vaccination has been widely recognised as a key public health achievement. However, vaccine hesitancy - bound up in uncertainty or lack of confidence in the safety and effectiveness of vaccination - may serve to undermine comprehensive coverage. Vaccine hesitancy is often examined on the scale of individual decision-making, or through referring to questions of ethics and freedom of choice. However, when considering the problem of vaccine hesitancy, social scientists of medicine would note that the public discourse surrounding vaccines – the way in which vaccines are represented and publicly understood - forms another important piece of the puzzle. It is not just individuals, but rather wider social representations of vaccination, which underpin vaccine hesitancy. Individual ‘choices’ are produced in the context of narratives and public conversations about the efficacy and safety of vaccine use. The ways in which vaccines are described and discussed in the public domain impacts upon private decision-making. Understanding these narratives is therefore pivotal to the wider understanding of the problem of vaccine hesitancy.

This paper examines the case study of the Council of Europe’s public criticism of mass vaccination during the H1N1 Influenza Pandemic. In December 2009, Council of Europe parliamentarian and epidemiologist Wolfgang Wodarg presented a recommendation to the Council of Europe entitled ‘Faked Pandemics: A Threat to Public Health’.^1^ Following subsequent months of debate, the Council of Europe passed a motion decrying the WHO’s public health reaction to H1N1. Criticism of the H1N1 vaccine was central to these findings. Such contestations can have an important effect on the public discourse, for example through media attention and public discussion of vaccination. The Council of Europe’s criticisms were a source of extended media commentary, and public scrutiny over the use of vaccines during the H1N1 pandemic continues. This paper draws upon social scientific analyses of vaccine narratives and illustrates the ways in which the Council of Europe’s account of H1N1 vaccines both reflected and influenced the wider societal discourses on vaccine uptake.

This paper utilises narrative analysis from the sociology of health to draw out the way in which vaccines were discussed and criticised. Narrative analysis allows for the identification of ideological and discursive constructions,^2^ including constructions surrounding the social ‘reality’ underpinning vaccine use. Narrative texts, as linguistic and discursive processes, are of fundamental importance in forming subjectivities and making the social world intelligible to those who live in it.^2^ The analysis of texts is important in understanding representations of disease, as narrative texts both present and constitute cultural interpretations of reality,^3^ providing insight into the social discourse underpinning this phenomena. The narratives and quotes discussed here are drawn out of a wider project which examined the global health management of the 2009-2010 H1N1 Pandemic. As part of this, a comprehensive analysis was made of all publicly available documents produced and published through the course of the Council of Europe’s discussions surrounding the WHO’s management of H1N1. This paper particularly utilises data gleaned from the textual analysis of expert testimony, parliamentary debates, parliamentary reports, and documents produced by the Council of Europe during their examination of the World Health Organization’s management of the H1N1 Pandemic, focussing on statements concerning the use or misuse of vaccines in combating H1N1.

Sociological analyses of vaccine resistance and hesitancy can be dated from Stern’s^4^ key early work in the sociology of vaccine uptake, which articulates the irrational motives, beliefs, and the vested interests of those who resist the use of vaccines. Critical to this field is the idea that, as Hobson-West^5^ rightly notes, vaccine resistance is a communal activity. Although vaccine resistance and hesitancy is made up of individual acts of refusal or uncertainty, resistance itself is found in the ‘anti-vaccination movements’, as structured social movements as well as contemporary social norms of ‘questioning’ vaccines.^6^ The voices of counter-vaccination can often be heard through the media, particular patient advocacy groups, and public discussions of the harms of immunization. Dew^7^ suggests that narratives surrounding vaccines which run counter to the public health discourse are generally rooted in scepticism of science, and may include: the fact that medical practice is held in suspicion due to inherent uncertainty and iatrogenesis; concerns over the competence, experience, or interest of individual medical practitioners, and; concerns over the side-effects of vaccination. The Council of Europe’s public discussion of the H1N1 vaccine contained elements of each of these common tropes. In particular, concerns over safety and side-effects formed substantial components of the arguments developed by the Council of Europe critics. However, additional arguments – which may also be seen as recurring themes within the counter-vaccine discourse – were also evident in this case.

The narratives that marked the case of the Council of Europe’s criticism of H1N1 vaccines were the following: 1.) Arguments surrounding trust and the decision to utilise mass vaccination as a public health tool in the case of H1N1; 2.) Criticisms of the efficacy of the vaccines themselves, including scepticism of the efficiency of vaccines in general, and the H1N1 vaccine in particular; and 3.) Questions surrounding the safety of these vaccines. Understanding these narratives can lead to a greater appreciation of ways in which the public health community can address vaccine hesitancy.

## Trust and Vaccination as an Effective Public Health Response

Scepticism or concern about vaccination is bound in problems of trust and mistrust. At the individual level, as Dew^7^ notes, this involves a particular patient’s trust in their medical practitioner, or in ‘medicine’ as a broader institution. The question of trust was also central to the Council of Europe’s contestation of H1N1 vaccination. In the case of the H1N1 pandemic, the Council of Europe argued that the World Health Organization was responsible for proposing vaccines as a management strategy. Rather than problematising trust in medicine or medical professionals, this case shows the problematisation of trust in public health structures and the World Health Organization.

For the WHO, influenza vaccination was understood as a pivotal public health strategy. Early mistrust in the H1N1 vaccines were regarded by the WHO as a problem given that, as the Special Advisor for Pandemic Influenza and Deputy Director-General Keiji Fukuda stated: ‘vaccines are really one of the prevention methods against infectious diseases which is best in terms of efficacy, [and] the safest’.^8^^,^ ^7^ Given concerns that, as one member of the press put it to Marie-Paul Keiny, WHO Director of the Initiative for Vaccine Research, ‘the [H1N1] vaccination campaign could actually create problems for the reputation of vaccines’^9^ the WHO strove to emphasise the safety and efficacy of H1N1 vaccines. The WHO upheld the dominant public health discourse surrounding the efficacy of vaccination.

The criticisms of the Council of Europe actors highlighted the problem of trusting the WHO. It was suggested by Paul Flynn (Council of Europe Rapporteur on the matter of the handling of H1N1), Hancock (representative for the UK), and Wodarg, that the WHO acted in an untrustworthy manner in having ‘cried wolf’^10^^,^ 11 ^,^ 12 over H1N1, and the ‘credibility’ and ‘accountability’ of the WHO had been undermined by the affair^1^^,^ 16, which was considered to be just one of ‘a whole series of scares’.^12^ These narratives were underpinned by the major political concern that the Council of Europe’s criticisms were addressing – that the mass vaccination campaign had been costly, since as Frahm (representative for Denmark) put it, the WHO’s recommendation had ‘forced countries to spend billions on unnecessary supplies’^10^ of antivirals and vaccines.

For the Council of Europe, the WHO’s decisions (and lack of credibility) had been a direct result of influence by pharmaceutical manufacturers. It was argued that the costly vaccination campaign had merely served to profit vaccine makers and that industry was able to ‘directly influence’ public health decisions surrounding H1N1.^11^ It was argued that there was ‘great commercial pressure’ to manage the H1N1 through the use of vaccines and other pharmaceuticals,^11^ and that ‘who pays the piper calls the tune’.^16^

Scepticism over the motives of key actors – the WHO and the producers of vaccines – was a key narrative. Action surrounding the H1N1 Pandemic reflected the globalised and rapid action surrounding the pandemic threat (as an urgent global problem, rather than scheduled vaccination regimes), and thus centred upon the role of the WHO. However, the issue of trust and accountability is more widely evident in issues of vaccine hesitancy. For instance, the Council of Europe actors’ scepticism was bound in concern of the greed and corruption of vaccine manufacturers. The concept of profit motives undermining the efficacy and safety of vaccines is recurring discourse in debates surrounding vaccine use.^17^ Building trust, not just in terms of scientific objectivity but in terms of wider institutional structures, is therefore fundamental to perceptions surrounding vaccination.

## The Efficacy of Vaccination

There is now a wide literature within the social sciences suggesting that the perception and management of risk is central to contemporary life at both the societal and the individual level.^18^^,^ 19 The notion of risk is particularly key to the issue of vaccine hesitancy – here, a lack of vaccine uptake is a choice (rather than a problem of lack of access), and this choice is underpinned by an understanding of the relative advantages and disadvantages of vaccine use. In assessing the benefits of vaccination, questions of efficacy are central. One of the ways in which vaccines are problematized is through challenging notions of efficacy, either at the general level or in regard to a particular type of vaccination. Such narratives of (in)efficacy were central to the Council of Europe actors’ representation of the H1N1 vaccination campaign.

The Council of Europe critics contested the need for administering vaccination against H1N1. The decision to undertake a mass immunisation campaign against the pandemic strain was decried as ‘without sufficient justification’ and unnecessary (Circene, representative for Latvia).^10^ This suggestion was reinforced – with the benefit of hindsight – by comparisons between countries that undertook vaccination and those that did not. So for example, it was argued that ‘[t]he country that spent the least was Poland, which rejected the idea that this disease was dangerous and which has suspicions about the safety of the vaccine...’ where, compared the Britain’s comparatively larger spending on pharmaceuticals ‘…the number of deaths per million from swine flu in Britain was about twice the number in Poland’ (Flynn).^10^ It was suggested that vaccination was therefore not an effective public health response to H1N1.

In addition to specific criticisms of the efficacy of the H1N1 vaccine, the critics also suggested that vaccination more generally does not act as an efficient public health measure. For example, Tom Jefferson from the Cochrane Institute was called in to provide expert testimony on the case. Dr Jefferson argued that ‘vaccines and antivirals have a weak or non-existent evidence base against influenza’.^14^ Such statements undermined the fundamental concept that influenza vaccination is an effective global health action. Likewise, it was argued that ‘the performance of the [influenza] vaccines in healthy adults is nothing to get excited about’ given that ‘we need to vaccinate 100 healthy adults to prevent one set of symptoms’.^14^ Other public health measures were described as having ‘a much better evidence base than vaccines’.^14^

The strategy of emphasising the lack of efficacy of vaccine use is a key narrative in problematizing vaccination. In the case of H1N1, it was suggested that vaccines were a costly and ineffective strategy against the pandemic. This uptake of narratives serves to minimise the ‘benefit’ aspect of the risk-to-benefit estimate that is central to estimations of the utility of vaccination.

## Vaccine Safety

Vaccine hesitancy at the individual level highlights the important role of the relative (perceived) weight of the risk of vaccination to the benefits incurred. Perceptions of the severity of the disease being vaccination against are weighed up against perceptions of adverse reactions to vaccination. Counter-vaccination movements are particularly prone to discourses of risk and uncertainty – vaccines are characterised as unsafe due to the risks of severe or lasting medical consequences. Unlike the short term and acute reactions acknowledged by the medical community, counter-vaccine narratives commonly emphasise long-term and severe reactions to vaccination.

Narratives that problematized vaccine safety were therefore central to the Council of Europe critics’ discussions. It was argued that ‘several independent medical experts raised warnings regarding excessive vaccination…[where]…there was no clinical scientific evidence to justify this’.^11^ The safety of vaccination was questioned and problematized.

In the case of H1N1, an important point of contention revolved around the novel manufacturing method, which was used, it was argued, due to the concern over quickening production and furthering profits. It was suggested that the vaccines ‘primarily follow[ed] economic strategies and was not to optimise public health needs’.^15^ This method, it was argued ‘involved higher risks than usual vaccines…[since]…some adjuvants were added and injected of which we know, that they stimulate the immune system manifold, which means that they could possibly lead to autoimmune diseases (such as multiple sclerosis) and immunological complication’.^15^

Several other potential adverse effects were cited. For example the ‘fast growing cancer-like cells’ were highlighted.^15^ While such statement were allusions rather than definitive statements, the problematisation of vaccine safety was evident in this account. It is also significant, because while (for example) allusions to the possible carcinogenic nature of the vaccines was not greatly emphasised within the Council of Europe’s hearing on the pandemic, such statements had been mobilised by the media and actors within the anti-vaccination movement,^15^^,^ 20 ^,^ 21 ^,^ 22 and may therefore inform wider perceptions about the safety of this vaccine and by extension, vaccines more generally.

## Conclusions

In order to understand the ways in which individuals make decisions regarding vaccine use, it is important to access the public discourse surrounding vaccination. Such narratives incorporate ideas about vaccinations which, while contrary to medical and scientific viewpoints, underpins the public understanding of vaccines. As such, it is important to see vaccine hesitancy as much a result of public discourse as it is a problem of individual decision-making.

The Council of Europe’s criticisms of the use of vaccines during the H1N1 Pandemic reflect many dominant discourses – lay understandings of vaccination and public representations of vaccination -that may inform vaccine hesitancy. Contestations such as this one, and the ways in which such debates are subsequently picked up by the media, have the potential to significantly shape the public discourse. Simultaneously, this debate mirrors and mobilises common sentiments surrounding vaccines.

Key to the Council of Europe’s account were three issues: the problem of trust in relation to the decision to use vaccines to combat H1N1, the problem of efficacy in terms of vaccines generally and the H1N1 vaccine specifically, and the problem of the risk posed by mass vaccination and the safety of the H1N1 vaccines. Examining the vaccine counter-narratives provides information that may be considered in addressing vaccine hesitancy. Trust building will be central to the task of impacting upon the public debate. Likewise, effective communication of issues of risk and trust – in ways that speak directly to the existing public understanding of vaccines – is also vital. Consequently it is important to acknowledge vaccine hesitancy as a problem not just of the individual level, but critically also of the social.

## Competing Interests

The authors have declared that no competing interests exist.
